# Novel vocalizations are understood across cultures

**DOI:** 10.1038/s41598-021-89445-4

**Published:** 2021-05-12

**Authors:** Aleksandra Ćwiek, Susanne Fuchs, Christoph Draxler, Eva Liina Asu, Dan Dediu, Katri Hiovain, Shigeto Kawahara, Sofia Koutalidis, Manfred Krifka, Pärtel Lippus, Gary Lupyan, Grace E. Oh, Jing Paul, Caterina Petrone, Rachid Ridouane, Sabine Reiter, Nathalie Schümchen, Ádám Szalontai, Özlem Ünal-Logacev, Jochen Zeller, Bodo Winter, Marcus Perlman

**Affiliations:** 1 Leibniz-Zentrum Allgemeine Sprachwissenschaft, 10117 Berlin, Germany; 2 Institut für deutsche Sprache und Linguistik, Humboldt-Universität zu Berlin, 10099 Berlin, Germany; 3 Institute of Phonetics and Speech Processing, Ludwig Maximilian University, 80799 Munich, Germany; 4 Institute of Estonian and General Linguistics, University of Tartu, 50090 Tartu, Estonia; 5 Laboratoire Dynamique Du Langage UMR 5596, Université Lumière Lyon 2, 69363 Lyon, France; 6 Department of Digital Humanities, University of Helsinki, 00014 Helsinki, Finland; 7 The Institute of Cultural and Linguistic Studies, Keio University, Mita Minatoku, Tokyo, 108-8345 Japan; 8 Faculty of Linguistics and Literary Studies, Bielefeld University, 33615 Bielefeld, Germany; 9 Department of Psychology, University of Wisconsin-Madison, Madison, WI 53706 USA; 10 Department of English Language and Literature, Konkuk University, Seoul, 05029 South Korea; 11 Asian Studies Program, Agnes Scott College, Decatur, GA 30030 USA; 12 Aix-Marseille Université, CNRS, Laboratoire Parole et Langage, UMR 7309, 13100 Aix-en-Provence, France; 13 Laboratoire de Phonétique et Phonologie, UMR 7018, CNRS & Sorbonne Nouvelle, 75005 Paris, France; 14 Department of Language and Communication, University of Southern Denmark, 5230 Odense, Denmark; 15 Department of Phonetics, Hungarian Research Centre for Linguistics, Budapest, 1068 Hungary; 16 School of Health Sciences, Department of Speech and Language Therapy, Istanbul Medipol University, 34810 Istanbul, Turkey; 17 School of Arts, Linguistics Discipline, University of KwaZulu-Natal, Durban, 4041 South Africa; 18 Department of English Language & Linguistics, University of Birmingham, Birmingham, B15 2TT UK

**Keywords:** Psychology, Evolution of language

## Abstract

Linguistic communication requires speakers to mutually agree on the meanings of words, but how does such a system first get off the ground? One solution is to rely on iconic gestures: visual signs whose form directly resembles or otherwise cues their meaning without any previously established correspondence. However, it is debated whether vocalizations could have played a similar role. We report the first extensive cross-cultural study investigating whether people from diverse linguistic backgrounds can understand novel vocalizations for a range of meanings. In two comprehension experiments, we tested whether vocalizations produced by English speakers could be understood by listeners from 28 languages from 12 language families. Listeners from each language were more accurate than chance at guessing the intended referent of the vocalizations for each of the meanings tested. Our findings challenge the often-cited idea that vocalizations have limited potential for iconic representation, demonstrating that in the absence of words people can use vocalizations to communicate a variety of meanings.

## Introduction

A key feature that distinguishes human language from other animal communication systems is its vast semantic breadth, which is rooted in the open-ended ability to create new expressions for new meanings. Many other animals use fixed, species-typical vocalizations to communicate information about their emotional state^1,2^ or to call out in alarm of specific classes of predators^3^, but only language serves for open reference of vocabulary to the myriad entities, actions, properties, relations, and abstractions that humans experience and imagine. This apparent gap presents a puzzle for understanding the emergence of language: how was human communication emancipated from the closed communication systems of other animals?

To get the first symbolic communication systems off the ground, our ancestors needed a way to create novel signals that could be understood by others prior to having in place any previously-established, *conventional* correspondence between the symbol and its meaning. Proponents of “gesture-first” theories of language origins have proposed one solution to this problem, hypothesizing that the first languages must have been rooted in visible gestures produced mainly with the hands^4–8^. Manual gestures are used by humans across cultures^9^, and have great potential for *iconicity*, that is, to communicate by resembling or directly cuing their meaning—such as in the parlor game “charades”. Gestures can pantomime actions, gauge the size and shape of entities, and locate referents in space^10^. Thus, they provide a widely applicable mechanism to enable communication between people who lack a shared vocabulary. Indeed, iconic gestures are known to play an important role in the emergence of new signed languages created in deaf communities^11^, as well as in the creation of symbolic home sign systems within families of deaf children and hearing adults^12^.

In comparison to gestures, the capacity for vocalizations to serve an analogous role in the emergence of spoken languages has remained largely the subject of speculation. An argument in favor of gesture-first theories is the operating assumption that vocalizations are severely limited in their potential for iconicity. Unlike the visual depiction enabled by gestures, the auditory modality does not easily allow the iconic representation of different kinds of concepts outside of the domain of sound^13–15^. Thus, unlike gestures, vocalizations cannot, to any significant degree, be used to facilitate communication between people who lack a shared vocabulary. This idea rests on the widely held and often-repeated claim that spoken language is primarily characterized by arbitrariness, where forms and meanings are related to each other only via conventional association^16^. The arbitrariness of spoken words is thought to be the result of an intrinsic constraint on the medium of speech^17^, with iconicity limited to the negligible exception of onomatopoeic words for sounds, such as *meow* (sound of a cat) and *bang* (sound of a sharp noise)^18,19^.

However, there is growing evidence that iconic vocalizations can convey a much wider range of meanings than previously supposed^20,21^. Experiments on sound symbolism—the mapping between phonetic sounds and meaning—demonstrate that people consistently associate different speech sounds, both vowels and consonants, with particular meanings^22,23^. Well documented cases include the “bouba–kiki” effect (e.g., rounded vowels and bilabial consonants associated with a round shape)^24,25^ and vowel–size symbolism (e.g., high front vowel /i/ associated with small size)^26^, but findings extend to mappings between speech sounds and various other properties as well (e.g., brightness, taste, speed, precision, personality traits)^22^. There is also increasing evidence for iconicity in the natural vocabularies of spoken languages^27,28^. Large-scale analyses of thousands of languages show that a large proportion of common vocabulary items carry strong associations with particular speech sounds, suggesting that the words in different languages may be motivated by correspondence to their meaning^29,30^. Cross-linguistic research also documents the widespread occurrence of ideophones (also called mimetics or expressives), a distinct class of depictive words used to express vivid meanings in semantic domains like sound (i.e., onomatopoeia), manner of motion, size, visual patterns, textures, and even inner feelings and cognitive states^31,32^. Notably, ideophones are, to an extent, understandable across cultures. Tested with words from five unfamiliar languages in a two-alternative comprehension task, Dutch listeners were more accurate than chance (50%) at selecting the meaning of ideophones, but modestly so^33^. They achieved a little above 65% accuracy with sound-related words, but only slightly above chance for the domains of color/vision, motion, shape, and texture. This moderate level of accuracy indicates a certain degree of iconicity in these words, but the ability to interpret them across cultures may be dampened by language-specific patterns in phonology, phonotactics, and morphology.

What remains to be assessed—and critical to the question of spoken language origins—is how well people are able to communicate across cultures with novel, non-linguistic vocalizations. Here we report the results of an extensive cross-cultural study to test whether people from diverse linguistic backgrounds can comprehend novel vocalizations created to express a range of meanings. Our stimuli included vocalizations for 30 different meanings that are common across languages and which might have been relevant in the context of early language evolution. These meanings spanned animate entities, including humans and animals (*child, man, woman, tiger, snake, deer*), inanimate entities (*knife, fire, rock, water, meat, fruit*), actions (*gather, cook, hide, cut, hunt, eat, sleep*), properties (*dull, sharp, big, small, good, bad*), quantifiers (*one, many*), demonstratives (*this, that*).

The stimuli were originally collected as part of a contest^34^. In the previous study, participants submitted a set of audio recordings of non-linguistic vocalizations, one for each meaning (no words permitted). The vocalizations were then evaluated by the ability of naïve listeners to guess their meaning from a set of written alternatives, with the winner determined as the set that was guessed most accurately. The vocalizations that were submitted reflected a variety of strategies, ranging from detailed, pantomime-like imitations (e.g., the sound of making a fire and then boiling water for *cook*) to more word-like vocalizations articulated with expressive prosody (e.g., *good* expressed with a nonce word spoken with rising tone, and *bad* a nonce word with a falling tone). Reduplication and repetition were common expressive elements, and within a set, many of the vocalizations bore systematic relationships to each other (e.g., a short simple sound articulated singly for *one* and multiply for *many*). Overall, guessing accuracy was well above chance for all contestants and across almost all of the meanings. However, all of the contestants who produced the vocalizations spoke English as a first or second language (with most living in the United States), and the comprehensibility of the vocalizations was assessed by American, English-speaking listeners.

**Figure 1:**
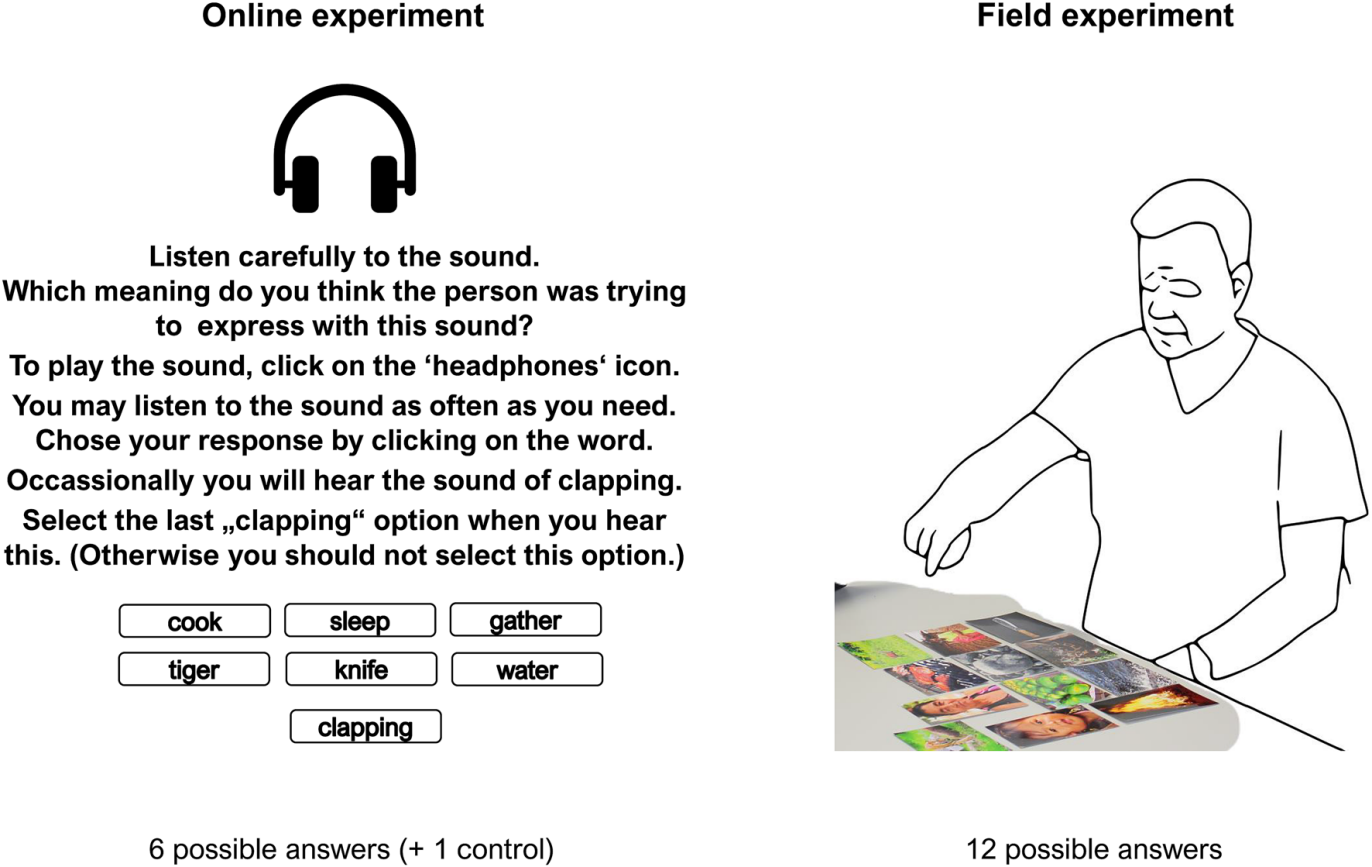
Experimental setup for online and fieldwork versions of the experiment.

The crucial test is to determine whether people from different cultural and linguistic backgrounds are able to infer the meanings of the vocalizations with similarly high levels of accuracy. To this end, we presented the recorded vocalizations—the three for each meaning that were guessed most accurately by American listeners—to diverse listeners in two cross-cultural comprehension experiments. In an online experiment with listeners of 25 different languages (from nine language families), participants listened to the 90 vocalizations (three for each of the 30 meanings), and for each, guessed its intended meaning from six written alternatives (the intended meaning plus 5 meanings selected randomly from the remaining 29). A second experiment was designed for participants living in oral societies with varying levels of literacy. In this version, which was conducted by an experimenter in the field, participants listened only to vocalizations for the 12 animate and inanimate entities that could be directly represented with pictures. They then guessed the intended referent, without the use of a written label, by pointing at one of 12 pictures—one for each referent—which were displayed in a grid in front of them. This experiment included three groups living in oral societies, along with four other groups for comparison. The setup of both online and fieldwork experiment is shown in Fig. 1. Altogether, these two experiments included speakers of 28 different languages spanning 12 different language families.

## Results

### Online experiment

**Figure 2:**
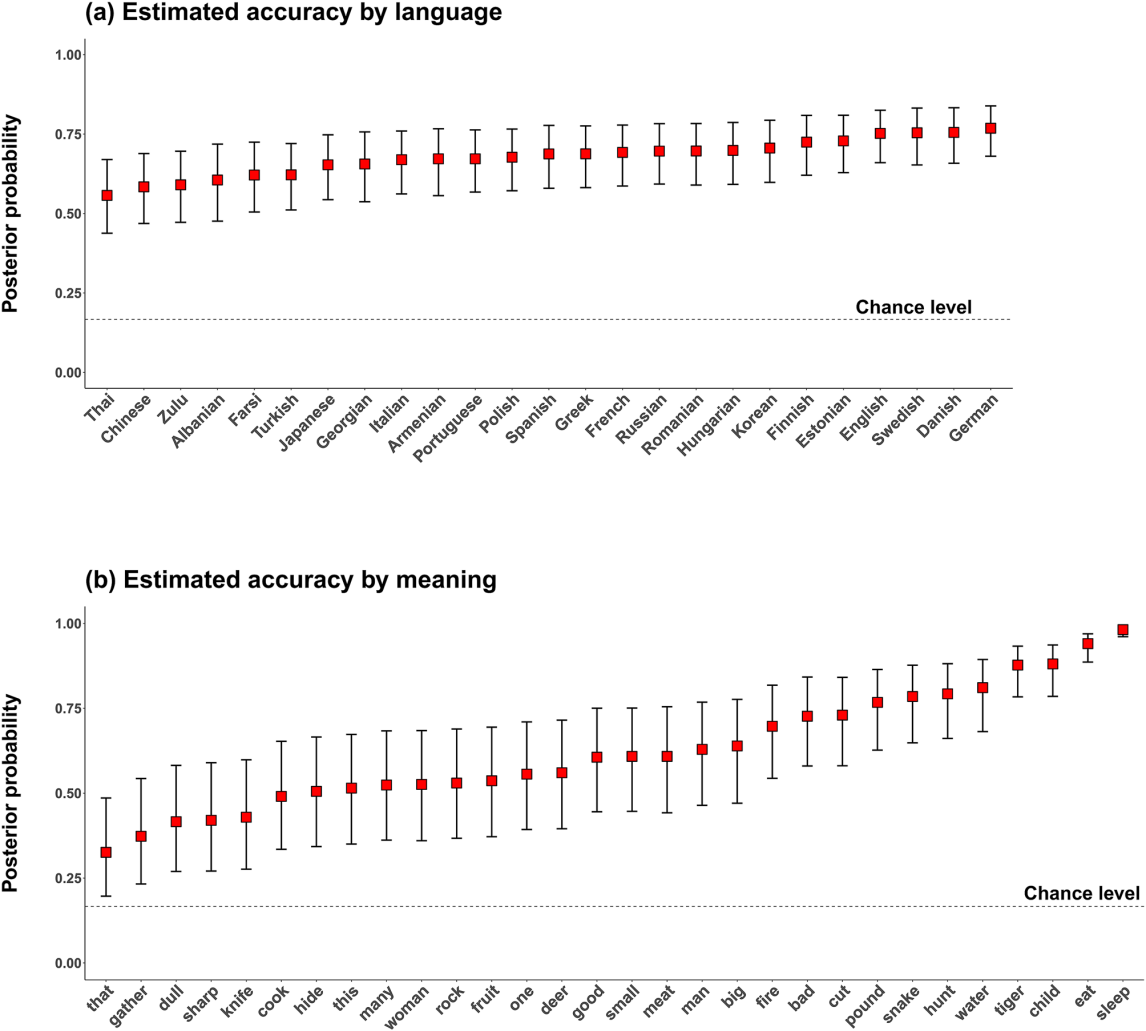
Posterior probability of a correct guess in the online experiment (**a**) per language and (**b**) concept; the red squares indicate the posterior means and error bars the 95% Bayesian credible intervals. The values displayed in this figure correspond to the model that takes into account random effect variation by meaning, vocalization, language, listener, and creator of vocalization.

First, we analyzed guessing accuracy by listeners of each language group with respect to the 30 different meanings. The overall average accuracy of responses across the 25 languages was 64.6%, much higher than chance level chance level (1/6 = 16.7%). The performance for each of the languages was above chance level, with means ranging from 52.1% for Thai to 74.1% for English. Across the board, a look at the descriptive averages shows that all meanings were guessed correctly above chance level, ranging from 34.5% (for the demonstrative *that*) to 98.6% (for the verb *sleep*) (Fig. 2b). For 20 out of the 25 languages performance was above chance for all meanings; for 4 out of 25 languages it was above chance for all but one meaning; and for one language, performance was correct for all but two meanings. Thus, all languages exhibited above-chance level performance for at least 28 out of the 30 meanings. A break-down of meaning categories shows that actions were guessed most accurately (70.9%), followed by entities (67.7%), properties (58.5%), and demonstratives (44.7%). If we look at nouns further, we see that vocalizations for animals were guessed most accurately (75.6%), followed by humans (69.9%), and inanimate entities (62.6%).

We fitted an intercept-only Bayesian mixed-effects logistic regression analysis to the accuracy data (at the single-trial level). The intercept of this model estimates the overall accuracy across languages. We controlled for by-listener, by-meaning, by-creator (i.e., the creator of the vocalization), and by-language family variation by fitting random intercept terms for each of these factors. In addition, we fitted a random intercept term for unique vocalizations (i.e., an identifier variable creating a unique label for each meaning x creator combination). This random intercept accounts for the fact that not just meanings and creators, but also particular vocalizations are repeated across subjects and hence are an important source of variation that needs to be accounted for. Controlling for all of these factors, the average accuracy was estimated to be 65.8% (posterior mean), with a 95% Bayesian credible interval spanning from 54.0 to 75.9%. The posterior probability of the average accuracy being below chance level is $p = 0.0$, i.e., not a single posterior sample is below chance. This strongly supports the hypothesis that across languages, people are able to reliably detect the meaning of vocalizations. Figure 2 displays the posterior accuracy means from the mixed model, demonstrating that performance was above chance level for all languages and for all meanings.

The mixed model also quantifies the variation in accuracy levels across the different random factors (see Table 1). High standard deviations in this table mean that accuracies differ more for this random effect variable. The meanings differ from each other most in accuracies (*SD* of logits $= 1.12$, 95% CI: [0.84, 1.50]), much more so than individual vocalizations (unique meaning-creator combinations, $SD = 0.56$, 95% CI: [0.46, 0.68]). Most notably, accuracy levels vary more across individual listeners ($SD = 0.33$, 95% CI: [0.30, 0.35]) than languages ($SD = 0.26$, 95% CI: [0.17, 0.37]), or language families ($SD = 0.24$, 95% CI: [0.02, 0.56]). This highlights the remarkable cross-linguistic similarity in the behavioral responses to this task: while there is variation across languages and language families, it is dwarfed by differences between listeners and differences between meanings.

**Table 1:** Random effects standard deviations from the Bayesian logistic regression model for the web data; ordered from highest to lowest standard deviation; larger SDs indicate that accuracy levels varied more for that random effect variable.

Random effect term	SD	95% Bayesian CI
Meaning	1.12	[0.84, 1.50]
Vocalization	0.56	[0.46, 0.68]
Listener	0.33	[0.30, 0.35]
Language	0.26	[0.17, 0.37]
Language family	0.24	[0.02, 0.56]
Creator of vocalization	0.13	[0.01, 0.40]

Because the vocalization stimuli were created by English speakers (either as a first or second language), we wanted to determine whether familiarity with English and related languages provided listeners with any advantage in understanding the vocalizations. To do this, first, we computed descriptive averages for accuracy as a function of people’s knowledge of English as either a first or second language. Our survey asked participants to note any second languages, and so we coded the data into four categories: English native speakers (‘English L1’, $N = 82$), participants who reported English as a second language (‘English L2’, $N = 648$), participants who reported another second language that was not English (‘non-English L2’, $N = 22$), and people who did not report to know any second language (‘no L2’, $N = 91$). All four groups performed on average much above chance level, with the English L1 speakers performing best (70.4%, descriptive average), followed by English L2 speakers (64.7%), ‘non-English L2’ speakers (60.1%), and ‘no L2’ speakers (59.7%). These descriptive averages suggest that there is a moderate advantage for English, but that listeners are still far above chance even without knowing English as a second language.

We also analyzed whether the genealogical similarity of listeners’ first language to English gave any guessing advantage. Indeed, speakers of Germanic languages (other than English) showed, on average, the highest accuracy (70.3%, descriptive average), compared to speakers who spoke a non-Germanic Indo-European language (63.2%), and speakers who spoke a non-Indo-European language (61.7%). This suggests a rough decline in accuracy given the genealogical distance of the speaker’s language to the language of the stimuli creators, although there is no difference between speakers of non-Germanic Indo-European languages and speakers from other language families.

### Field experiment

**Figure 3:**
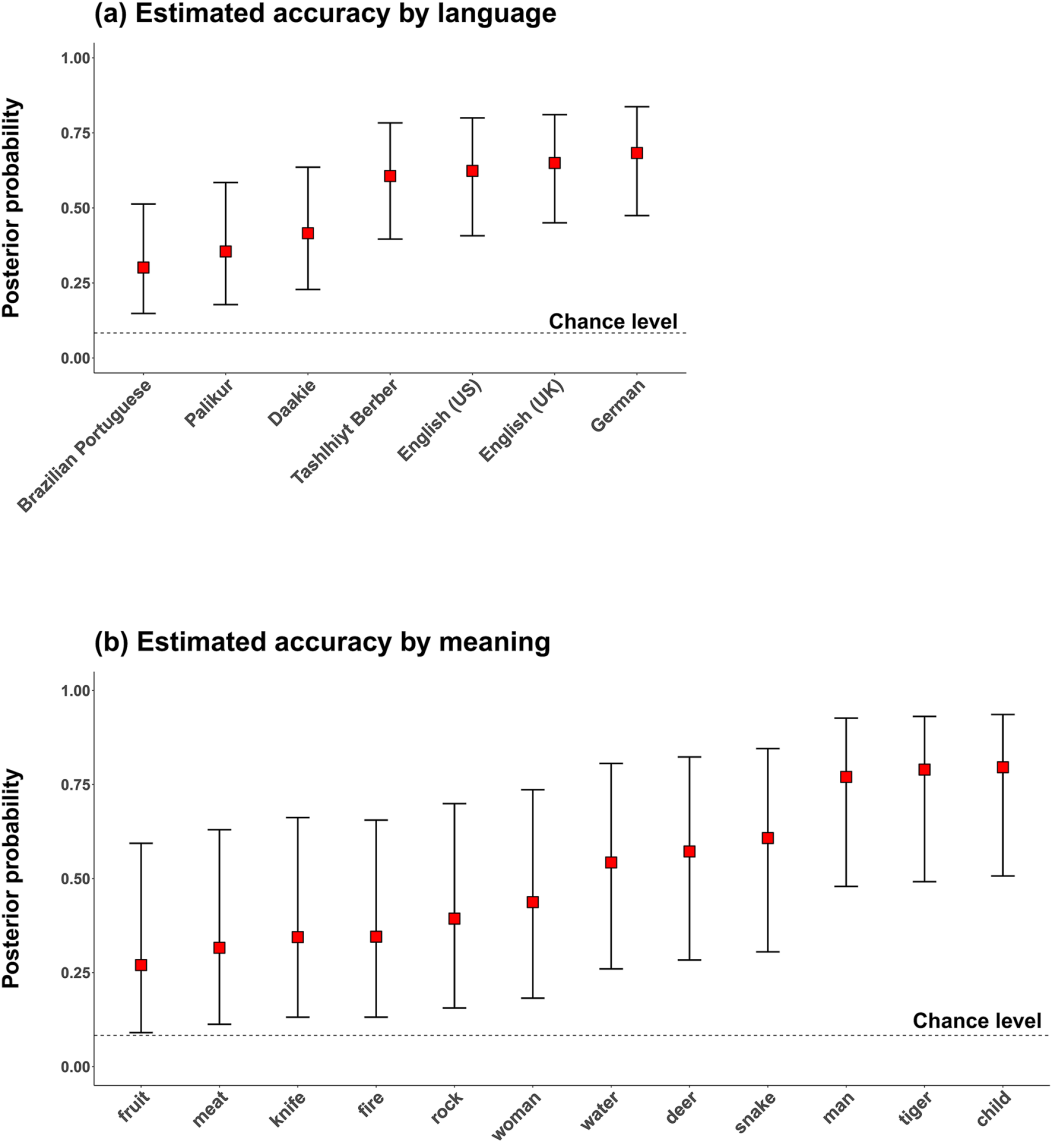
Posterior probability of a correct guess in the field experiment (**a**) per language and (**b**) concept; the red squares indicate the posterior means and error bars the 95% Bayesian credible intervals. The values displayed in this figure correspond to the model that takes into account random effect variation by meaning, vocalization, language, listener, and creator of vocalization.

First, we analyzed guessing accuracy for the seven language groups for the 12 meanings. Note that “language group” is defined in the field experiment as a unique socio-political population speaking a given language; for our data, the groups map uniquely to the languages, with the exception of the distinction between American English and British English speakers, which maps to groups. On average, responses across the language groups were correct 55.0% of the time, much above chance level ($1/12 = 8.3\%$, as there were 12 possible answers, among which one was correct). Listeners of all languages performed above chance, ranging from 33.8% (Portuguese speakers in Brazilian Amazonia) to German (63.1%). All meanings were guessed above chance level, ranging from 27.5% (*fruit*) to 85.5% (*child*) (Fig. 3b). Five of the language groups exhibited above-chance level performance for all 12 meanings, and the remaining two groups—Brazilian Portuguese and Palikúr speakers—exhibited above-chance level performance for 10 out of the 12 meanings.

As before, we used an intercepts-only Bayesian logistic regression model to analyze this data, with random effects for vocalization (unique meaning-creator combination), meaning, creator of vocalization, listener, and language group. While controlling for all these factors, the model estimated the overall accuracy to be 51.9%, with the corresponding Bayesian 95% credible spanning from 26.3 to 75.9%, thus not including chance-level performance ($1/12 = 8.3\%$). The posterior probability of the accuracy mean being below chance for the field sample was very low ($p < 0.0001$), thus indicating strong evidence for above-chance level performance across languages. Figure 3 displays the posterior means for languages and meanings from this model.

A look at the random effects (see Table 2) shows that, similarly to the online experiment, accuracy variation was biggest for meaning ($SD = 1.11$), followed by vocalization (unique meaning-creator combination; $SD = 0.94$). In contrast to the online experiment, variation across languages ($SD = 0.87$) was higher than variation across listeners ($SD = 0.57$). This may reflect the cultural and linguistic heterogeneity of this sample, compared to the online experiment.

**Table 2:** Random effects standard deviations from the Bayesian logistic regression model for the field data; ordered from highest to lowest standard deviation; larger SDs indicate that accuracy levels varied more for that random effect variable.

Random effect term	SD	95% Bayesian CI
Meaning	1.11	[0.53, 1.95]
Vocalization	0.94	[0.67, 1.31]
Language	0.87	[0.43, 1.81]
Listener	0.57	[0.47, 0.69]
Creator of vocalization	0.27	[0.01, 0.91]

Finally, we assessed whether vocalizations for animate entities were guessed more accurately than inanimate entities. As the animate entities—three human and three animal—produce characteristic vocal sounds, these referents may be more widely recognizable across language groups. On average, responses were much more likely to be correct for animate entities (72%) than inanimate entities (39%). To assess this inferentially, we amended the above-mentioned Bayesian logistic regression analysis with an additional fixed effect for animacy. The model indicates strong support for animate entities being more accurate across languages: the odds of these being more accurate were 5.10 to 1 (log odd coefficient: $1.63, SE = 0.49$), with the Bayesian 95% credible interval for this coefficient ranging from an odds of 1.8 to 13.2. The posterior probability of the animacy effect being below chance level is $p = 0.0$ (not a single posterior sample was below zero for this coefficient), which supports a cross-linguistic animacy effect.

## Discussion

Can people from different cultures use vocalizations to communicate, independent of the use of spoken words? We examined whether listeners from diverse linguistic and cultural backgrounds were able to understand novel, non-linguistic vocalizations that were created to express 30 different meanings (spanning actions, humans, animals, inanimate entities, properties, quantifiers, and demonstratives). Our two comprehension experiments, one conducted online and one in the field, reached a total of 986 participants, who were speakers of 28 different languages from 12 different families. The online experiment allowed us to test whether a large number of diverse participants around the world were able to understand the vocalizations. With the field experiment, by using the 12 easy-to-picture meanings, we were able to test whether participants living in predominantly oral societies were also able to understand the vocalizations. Notably, in this task, participants made their response without the use of written language, by pointing to matching pictures. In both experiments, with just a few exceptions, listeners from each of the language groups were better than chance at guessing the intended referent of the vocalizations for all of the meanings tested.

The stimuli in our experiments were created by English-speakers (either as a first or second language), and so to assess their effectiveness for communicating across diverse cultures, it was important to determine specifically whether the vocalizations were understandable to listeners who spoke languages that are most distant to English and other Indo-European languages. In the online experiment, we did find that native speakers of Germanic languages were somewhat more accurate in their guessing compared to speakers of both non-Indo-European and non-Germanic Indo-European languages. We also found that those who knew English as a second language were more accurate than those who did not know any English. However, accuracy remained well above chance even for participants who spoke languages that are not at all, or only distantly, related to English.

In the field experiment, while guessing exceeded chance across the language groups for the great majority of meanings, we did observe, broadly, two levels of accuracy between the seven language groups. This difference primarily broke along the lines of participant groups from predominantly oral societies in comparison to those from literate ones. The Tashlhiyt Berber, United States and British English, and German speakers all displayed a similarly high level of accuracy, between 57 and 63% ($\hbox {chance} = 1/12 = 8.4\%$). In comparison, speakers of Brazilian Portuguese, Palikúr, and Daakie were less accurate, between 34 and 43%. Notably, the Berber group, like the English and German groups, consisted of university students, whereas listeners in the Portuguese, Palikúr, and Daakie groups, living in largely oral societies, had not received an education beyond a primary or secondary school. This suggests that an important factor in people’s ability to infer the meanings of the vocalizations is education^35^, which is known to improve performance on formal tests^36^. A related possibility is that the difference between the oral and literate groups is the result of differential experience with wider, more culturally diverse social networks, such as through the Internet.

Not surprisingly, we found, even across our diverse participants, that some meanings were consistently guessed more accurately than others. In the online experiment, collapsing across language groups, accuracy ranged from 98.6% for the action *sleep* to 34.5% for demonstrative *that* ($\hbox {chance} = 1/6 = 16.7\%$). Participants were best with the meanings *sleep, eat, child, tiger*, and *water*, and worst with *that, gather, dull, sharp*, and *knife*, for which accuracy was, statistically, just marginally above chance. In the field experiment, accuracy ranged from 85.5% for *child* to 27.5% for *fruit*. Across language groups, animate entities were guessed more accurately than inanimate entities.

There is not a clear benchmark for evaluating the accuracy with which novel vocalizations are guessed across cultures. However, an informative point of comparison is the results of experiments investigating the cross-cultural recognition of emotional vocalizations—an ability that has been hypothesized to be a psychological universal across human populations. For example, Sauter and colleagues^2^ showed that Himba speakers living in Namibian villages, when presented with vocalizations for nine different emotions produced by English speakers, identified the correct emotion from two alternatives with 62.5% accuracy, with identification reliably above chance for six of the nine emotions tested (i.e., for the basic emotions). More generally, a recent meta-analysis found that vocalizations for 25 different emotions were identifiable across cultures with above-chance accuracy, ranging from about 67% to 89% accuracy when scaled relative to a 50% chance rate, although the number of response alternatives varied across the included studies^37^. This analysis found an in-group advantage for recognizing each of the emotions, with accuracy negatively correlated with the distance between the expresser and perceiver culture (also see^38^). Considering these studies, the recognition of emotional vocalizations appears roughly comparable to the current results. But critically, in comparison to the single domain of emotions, iconic vocalizations can enable people from distant cultures to share information about a far wider range of meanings. Although the use of discrete response alternatives in a forced-choice task presents a greatly simplified context for communication, our use of 6 alternatives in the online experiment and 12 in the field experiment is considerably expanded from the typical two alternative tasks used in many cross-cultural emotion recognition experiments.

Thus, our study indicates the possibility that iconic vocalizations could have supported the emergence of an open-ended symbolic system. While this undercuts a principal argument in favor of a primary role of gestures in the origins of language, it raises the question of how vocalizations would compare to gestures in a cross-cultural test. Laboratory experiments in which English-speaking participants played charades-like communication games have shown better performance with gestures than with non-linguistic vocalizations, although vocalizations are still effective to a significant extent^39,40^. In this light, we highlight that while our findings provide evidence for the potential of iconic vocalizations to figure into the creation of the original spoken words, they do not detract from the hypothesis that iconic—as well as indexical—gestures also played a critical role in the evolution of human communication, as they are known to play in the modern emergence of signed languages^11^. The entirety of evidence may be most consistent with a multimodal origin of language, grounded in iconicity and indexical gestures^21,41^. Both modalities, visual and auditory, could work together in tandem, each better suited for communicating under different environmental conditions (e.g., daylight vs. darkness), social contexts (e.g., whether the audience is positioned to better see or hear the signal), and the meaning to be expressed (e.g., animals, actions, emotions, abstractions).

An additional argument put forward in favor of gestures over vocalizations in language origins is the long-held view that great apes—our closest living relatives including chimpanzees, gorillas, and orangutans—have far more flexible control over their gestures than their vocalizations, which are limited to a species-typical repertoire of involuntary emotional reflexes (e.g.,^6,15,42–44^). Yet, recent evidence shows that apes do in fact have considerable control over their vocalizations, which they produce intentionally according to the same criteria used to assess the intentional production of gestures^45^. Studies of captive apes, especially those cross-fostered with humans, show apes can flexibly control pulmonary airflow in coordination with articulatory movements of the tongue, lips, and jaw, and they are also able to exercise some control over their vocal folds^46,47^. Given these precursors for vocal dexterity in great apes, it appears that early on in human evolution, an improved capacity to communicate with iconic vocalizations would have been adaptive alongside the use of iconic and indexical gestures. Thus, there would have been an immediate benefit to the evolution of increased vocal control (e.g., increased connections between the motor cortex and the primary motor neurons controlling laryngeal musculature^48^)—one that would be much more widely applicable than the modulation of vocalizations for the expression of size and sex (e.g.,^49^).

Altogether, our experiments present striking evidence of the human ability to produce and understand novel vocalizations for a range of meanings, which can serve effectively for cross-cultural communication when people lack a common language. The findings challenge the often-cited idea that vocalizations have limited potential for iconic representation^13–19^. Thus, our study fills in a crucial piece of the puzzle of language evolution, suggesting the possibility that all languages—spoken as well as signed—may have iconic origins. The ability to use iconicity to create universally understandable vocalizations may underpin the vast semantic breadth of spoken languages, playing a role similar to representational gestures in the formation of signed languages.

## Methods

### Online experiment

#### Participants

The online experiment comprises a sample totaling 843 listeners (594 women, 193 men; age 18–84, mean age: 32) from 25 different languages. This is after we excluded speakers who did not perform accurately on at least 80% of the control condition trials (10 repetitions of the clapping sound in total), and who did not complete at least 80% of the experiment. For 38 US speakers, we failed to ascertain gender and age information. The languages span nine different language families: Indo-European, Uralic, Niger-Congo, Kartvelian, Sino-Tibetan, Tai-Kadai, and the language isolates Korean and Japanese. On average, there were about 33 listeners per language, ranging from seven for Albanian to 77 for German. Table 3 shows the number of speakers per language in the online sample. The sample was an opportunity sample that was assembled via snowballing. We used our own contacts and social media, asking native speakers of the respective languages to share the survey.

All participants declared that they took part in the experiment voluntarily. The informed consent was obtained from all participants and/or their legal guardians. The experiment was a part of a project that was approved by the ethics board of the German Linguistic Society and the data protection officer at Leibniz-Centre General Linguistics. The experiment was performed in accordance with the guidelines and regulations provided by the review board.

**Table 3:** Number of listeners and average accuracy (descriptive averages, chance = 16.7%) for each language in the sample for the online experiment in alphabetical order by language family and genus.

Family	Genus	Name	N of listeners	Accuracy (%)
Altaic	Turkic	Turkish	38	58
Indo-European	Albanian	Albanian	7	54
	Armenian	Armenian	16	63
	Germanic	English	82	74
		German	77	72
		Swedish	18	72
		Danish	18	71
	Hellenic	Greek	36	64
	Indo-Iranian	Farsi	20	57
	Romance	French	51	65
		Romanian	25	65
		Spanish	34	64
		Portuguese	55	63
		Italian	52	63
	Slavic	Russian	32	65
		Polish	48	63
Japanese	Japanese	Japanese	46	61
Kartvelian	Kartvelian	Georgian	11	63
Korean	Korean	Korean	20	66
Niger-Congo	Bantoid	Zulu	18	55
Sino-Tibetan	Chinese	Mandarin	32	55
Tai-Kadai	Kam-Tai	Thai	15	52
Uralic	Finnic	Estonian	43	68
		Finnish	16	68
	Ugric	Hungarian	32	65

Within a genus, the languages are sorted by the accuracy (see Fig. 2 for complementary posterior estimates and 95% credible intervals from the main analysis).

#### Stimuli

The stimuli were collected as part of a contest in a previous study^34^. Participating contestants submitted a set of vocalizations to communicate 30 different meanings spanning actions (*sleep, eat, hunt, pound, hide, cook, gather, cut*), humans (*child, man, woman*), animals (*snake, tiger, deer*), inanimate entities (*fire, fruit, water, meat, knife, rock*), properties (*big, small, sharp, dull*), quantifiers (*many, one*), and demonstratives (*this, that*).

To choose the winner of the contest, Perlman and Lupyan (2018)^34^ used Amazon Mechanical Turk to confront native speakers of American English with the submitted vocalizations, and asked them what the intended meaning of each vocalization was. In the current study, a subset of the vocalizations initially submitted to the contest was used. For each meaning, the three most accurately guessed vocalizations from Perlman and Lupyan’s (2018)^34^ evaluation were chosen, regardless of the submitting contestant. The recordings can be found in the Open Science Framework repository: https://osf.io/4na58/.

#### Procedure

Participants listened to each of the 90 vocalizations, and for each one, guessed its meaning from 6 alternatives (the correct meaning plus five randomly generated alternatives).

The surveys were translated from English into each language by native speakers of the respective languages. The translation sheet consisted of an English version to be translated and additional remarks for possibly ambiguous cases. In such cases, the literal meaning was the preferred one. The online surveys were hosted with Percy, an independent online experiment engine^50^. All surveys posted online were checked by the first author and by the respective translators and/or native speakers prior to distribution.

The surveys included a background questionnaire in which the participants were asked to report the following information: their sex, age, country of residence, native language(s), foreign language(s), if they spoke a dialect of their native language, and the place where they entered primary school. In addition, they were asked about their hearing ability, the environment they were in at the time of taking the survey, the audio output device, and the input device. After completing the background questionnaire, the participants proceeded directly to the experimental task.

In each of the language versions, the task was the same. The participants listened to a vocalization and were asked to guess which meaning it expressed from among six alternatives—five of which were generated randomly from a pool of other not matching meanings. To check whether the participants were paying attention, ten control trials were added in which the sound of clapping was played. The participants were instructed to choose an additional option—“clapping”—for these trials. The presentation order was randomized for each participant. The sound could be played as many times as a participant wanted. Participants played the sound on average 1.64 times (a single sound $= 61.6\%$ of all trials, 1 repetition $= 25.6\%$ of all trials).

#### Statistical analysis

All analyses were conducted with R^51^. We used the tidyverse package for data processing^52^ and the ggplot2 package for Figs. 2 and 3^53^. All analyses can be accessed via the Open Science Framework repository (https://osf.io/4na58/).

We fitted Bayesian mixed logistic regression models with the brms package^54^ for the online experiment and field experiment data separately. These models were intercept-only to estimate the overall accuracy level. The mixed-effects model included random intercepts for listener ($N = 843$), creator of the vocalization (11 creators), meaning (30 meanings), unique vocalization (30 × 3, unique meaning-creator combination), language (25 languages), language family (nine families, classifications from the Autotyp database^55^), and language area (7 macro areas from Autotyp).

MCMC sampling was performed using Stan and the wrapper function brms^54^ with 4000 iterations for each of 4 chains (2000 warm-up samples), resulting in a total of 8000 posterior samples. There were no divergent transitions and all ${\hat{R}}$ values were $= 1.0$, indicating successful convergence. Posterior predictive checks indicated that the posterior predictions approximate the data well.

### Field experiment

#### Participants

We analyzed data from 143 listeners (99 female; 44 male; average age 28; age range 19–75) who were speakers of six different languages, including Tashlhiyt Berber, Daakie, Palikúr, Brazilian Portuguese, two dialects of English (British English and American English), and German. Thus, this set comprised four groups from Indo-European languages (Brazilian Portuguese, English, and German), and three groups from different language families: Afro-Asiatic (Berber), Arawakan (Palikúr), and Austronesian (Daakie). Notably, many of the participants in these groups spoke multiple languages. For example, the Tashlhiyt Berber group consisted of university students who studied in French, and the listeners from predominantly oral cultures (speakers of Daakie, Palikúr, and Portuguese) also had some knowledge of other languages spoken in the respective regions. As shown in Table 4, the number of listeners range from seven listeners for Palikúr to 56 listeners for British English, with a mean of 20 listeners per language.

For each of the respective languages, the experiments were conducted on-site. This included the University Ibn Zohr in Agadir, Morocco (Tashlhiyt Berber), Port Vato on the South Pacific island of Ambrym, Vanuatu (Daakie), the banks of Oyapock river near St. Georges de l’Oyapock in French Guayana (Palikúr), Cametá in Brazil (Brazilian Portuguese), the psychology laboratory at Madison in the US (American English), Birmingham in the UK (British English), and Berlin as well as the Baltic Sea region in Germany (German).

#### Stimuli

For this experiment, we used vocalizations for the twelve animate and inanimate entities only, which could be well displayed in pictures. These included: *child, deer, fire, fruit, knife, man, meat, rock, snake, tiger, water*, and *woman*. The pictures depicted the meanings as simply as possible, with preferably no other objects in sight. They were chosen from a flickr.com database under the Creative Commons license. For each meaning, the stimuli included the same vocalizations that were part of the online experiment, so that there were 36 vocalizations presented as auditory stimuli (12 meanings × 3 creators).

**Table 4:** Number of listeners and average accuracy (descriptive averages, chance = 8.3%) for each language in the sample for the field experiment; in alphabetical order by language family.

Family	Genus	Name	Location	N of listeners	Accuracy (%)
Afro-Asiatic	Berber	Tashlhiyt Berber	Agadir, Morocco	20	57
Arawakan	Eastern Arawakan	Palikúr	Saint-Georges-de-l’Oyapock, French Guyana	7	37
Austronesian	Oceanic	Daakie	Port Vato, Ambrym, Vanuatu	12	43
Indo-European	Germanic	German	Berlin, Germany; Baltic Sea region, Germany	19	63
		English (UK)	University of Birmingham, UK	56	60
		English (US)	University of Wisconsin, USA	16	59
	Romance	Brazilian Portuguese	Cametá, Pará, Brazil	13	34

Within a language family, the languages are sorted by the accuracy (see Fig. 3 for complementary posterior estimates and 95% credible intervals from the main analysis).

#### Procedure

The pictures of the 12 referents were placed on the table in front of the participant, and their order was pre-randomized in four versions. The experimenter—naïve to the intended meaning of a sound file—played the vocalizations in a pre-randomized order, also in four order versions. For each sound, the listener was asked to choose a picture that depicted the sound best from among the pictures lying in front of her/him. The exact instructions given in the native language of the participant were:

"You will hear sounds. For each sound, choose a picture that it could depict. More than one sound can be matched to one picture".

Participants made their response by pointing at one of the twelve photos laid out in a grid before them. The experimenter noted the answers on an anonymized response sheet. During the experiment, the experimenter was instructed to look away from the pictures in order to minimize the risk of giving the listener gaze cues. The sound could be played as many times as the participant wanted.

The background questionnaire for the field version was filled out by the experimenter and consisted of questions for: sex, age, native language(s), and other language(s).

The data were collected in various locations (cf. “Participants” above) and therefore also in slightly different conditions. While the American, British, and Berber participants were recorded in a university room, the case was different for the remote populations. German speakers were partly recorded in a laboratory room (Berlin), partly in a quiet indoor area at the Baltic seacoast. The Daakie participants were recorded in a small concrete building of the Presbyterian Church; participants were seated on a bench in front of a table, and an attempt was made that they were not disturbed by curious onlookers. Brazilian Portuguese listeners in Cametá were recorded in their own houses, and Palikúr speakers in a community building in Saint-Georges-de-l’Oyapock where they were interviewed individually in a separate room.

#### Statistical analysis

The mixed model included random intercepts for meaning (12 different meanings), creator of the vocalization (7 creators), unique vocalization (36 unique meaning–creator combination), listeners (143 listeners), and language (6 languages). We used a uniform prior (− 10, 10) on the intercept for both the field and online experiment. To assess the effect of animacy, an additional model was fitted for the field experiment with animacy as a fixed effect and a regularizing normal prior ($\mu = 0, \sigma = 2$), as well as with by-language and by-speaker varying animacy slopes.
